# Underrepresentation of ethnic minorities in UK COVID-19 trials: comment on a recent systematic review and meta-analysis

**DOI:** 10.1186/s12916-023-03002-6

**Published:** 2023-08-15

**Authors:** Zeynep Ersoy Guller, Frederick Green, Anna L. Goodman

**Affiliations:** Guy’s and St Thomas’ NHS Foundation Trust, London, UK

**Keywords:** COVID-19, Ethnicity, Diversity, Clinical trials, Medical research

## Background

During the Coronavirus disease (COVID-19) pandemic, clinical trials investigating potential vaccines and therapeutics have led to incredible breakthroughs and greatly improved outcomes; however, there have been growing concerns regarding the underrepresentation of ethnic minorities among trial participants [1]. This is concerning for two primary reasons. First is to ensure that trial interventions are actually effective in the target population, with COVID-19 known to disproportionately affect ethnic minorities [2]. Second is a lack of representation fuels mistrust in clinical trials [3], which may lead to vaccine hesitancy in minority communities, resulting in worse outcomes from COVID-19 infection.

A recent systematic review and meta-analysis by Murali et al. in *BMC Medicine* [4] revealed an underrepresentation of ethnic minorities in UK-based COVID-19 clinical trials and made suggestions to help improve ethnic minority representation in future trials.

## Comparison to 2021 ONS Census

Murali et al. reported on the representation of different ethnicities in UK-based COVID-19 clinical trials, using the ONS 2011 Census figures as a reference. However, the recent publication of the ONS 2021 Census alters the interpretation [5].

Across 29 trials, their meta-analysis revealed 84.8% [95% confidence interval (CI) 81.6–87.5%] of participants were White, leading the authors to suggest this group were under-represented when compared to the ONS 2011 figure of 86%. However, when compared to the updated ONS 2021 Census figure of 81.7%, this group should be classified as over-represented.

In addition, Black, Asian and Mixed participants are likely more under-represented than reported by Murali et al., if compared to the ONS 2021 Census. For example, their meta-analysis of 17 trials revealed that 1% [95% CI 0.6–1.5%] of participants were Black, which they compared to the ONS 2011 Census figure of 3.3% to show they were under-represented. The ONS 2021 Census showed that 4% of the population are classified as Black, suggesting the group are in fact more under-represented than reported. Similar patterns are seen for Asian and Mixed participants. These updated results confirm Murali et al.’s first interpretation of their data, that “ethnic minority groups are more likely to be under-represented in COVID-19 trials” [4].

Furthermore, the ONS 2021 Census has changed the interpretation for the Other group. Murali et al.’s meta-analysis of 17 trials showed that 1.7% [95% CI 1.1–2.8%] of participants were Other, which they compared to the ONS 2011 figure of 1%, suggesting the group were being over-represented. This led Murali et al. to suggest a possible second interpretation of their data, that “Asian, Black and Mixed ethnicities were more likely to be classified as ‘Other’” and that this “raises questions about data accuracy and reporting”. However, the ONS 2021 Census showed the Other group now represent 2.1% of the population, meaning they are likely under-represented in the clinical trials reviewed by Murali et al. This updated analysis would counter their second interpretation.

## Choice of a reference population

Of the 17 studies that reported on the proportion of participants that were Black, nine were vaccine trials and eight were therapeutic trials. The five studies with the highest proportion of Black participants were all therapeutic trials and four of the five studies with the lowest proportion of Black participants were vaccine trials. These trends can likely be explained by the disproportionate effect of COVID-19 on ethnic minorities [2], creating increased opportunity for recruitment into therapeutic trials. As a result, it may not be appropriate to use the ONS Census data as a reference in COVID-19 therapeutic trials as this may not show the true scale of underrepresentation of ethnic minorities.

A similar systematic review and meta-analysis by Xiao et al. looked at ethnic representation in US-based COVID-19 trials [6]. They instead used the “US population diagnosed with COVID-19” as a reference, suggesting it is “a more appropriate determinant of representation across demographic groups than the general US population” [6]. If a similar measure was used by Murali et al., it would likely have shown a greater underrepresentation of ethnic minority groups in therapeutic trials in the UK.

## Vaccine uptake: the cause of excess mortality?

The trials that were included in Murali et al.’s meta-analysis enrolled patients between March 2020 and November 2021, corresponding to the first, second and Alpha waves of the pandemic [7]. During the first wave of the pandemic (January 2020—September 2020) the COVID-19 death rate was highest in the Black African group [8]. During the second wave (September 2020—January 2021), which also included the start of the vaccination programme in December 2020, COVID-19 mortality was highest in Bangladeshi ethnic group, followed by Pakistani and Black African groups [9] with similar figures during the Alpha period (January 2021-June 2021). In a recent population-based cohort study published in *BMC Medicine,* Bosworth et al. showed that although geography, socioeconomic factors, and pre-existing health conditions explained the majority of the elevated COVID-19 mortality risk in adjusted models, lower vaccination uptake in the Black African, Black Caribbean, and Pakistani groups may have driven some of the differences in COVID-19 mortality compared to White British [10]. This implies that any measures that aim to reduce vaccine hesitancy in ethnic minority groups, including ensuring good representation in clinical trials, may have an impact on outcomes from COVID-19 infection.

## Conclusions

Murali et al.’s systematic review and meta-analysis shone a light on the important issue of underrepresentation of ethnic minority groups in COVID-19 clinical trials in the UK. We agree that mistrust and vaccine hesitancy are complex problems and hope that Murali et al.’s suggestions will help improve ethnic minority representation in future trials.

## Data Availability

Not applicable.

## Abbreviations

CI: Confidence Interval
COVID‑19: Coronavirus disease
ONS: Office for National Statistics Census
UK: United Kingdom
US: United States
